# Divergence of thermal physiological traits in terrestrial breeding frogs along a tropical elevational gradient

**DOI:** 10.1002/ece3.2929

**Published:** 2017-04-06

**Authors:** Rudolf von May, Alessandro Catenazzi, Ammon Corl, Roy Santa‐Cruz, Ana Carolina Carnaval, Craig Moritz

**Affiliations:** ^1^ Department of Ecology and Evolutionary BiologyMuseum of ZoologyUniversity of MichiganAnn ArborMIUSA; ^2^ Museum of Vertebrate ZoologyUniversity of California, BerkeleyBerkeleyCAUSA; ^3^ Department of ZoologySouthern Illinois University CarbondaleCarbondaleILUSA; ^4^ Área de HerpetologíaMuseo de Historia Natural de la Universidad Nacional de San Agustín (MUSA)ArequipaPerú; ^5^ Department of BiologyThe City University of New YorkNew YorkNYUSA; ^6^ Centre for Biodiversity Analysis and Research School of BiologyThe Australian National UniversityCanberraACTAustralia

**Keywords:** Amazon, Andes, critical thermal limits, CT_max_, CT_min_, physiological divergence

## Abstract

Critical thermal limits are thought to be correlated with the elevational distribution of species living in tropical montane regions, but with upper limits being relatively invariant compared to lower limits. To test this hypothesis, we examined the variation of thermal physiological traits in a group of terrestrial breeding frogs (Craugastoridae) distributed along a tropical elevational gradient. We measured the critical thermal maximum (CT_max_; *n* = 22 species) and critical thermal minimum (CT_min_; *n* = 14 species) of frogs captured between the Amazon floodplain (250 m asl) and the high Andes (3,800 m asl). After inferring a multilocus species tree, we conducted a phylogenetically informed test of whether body size, body mass, and elevation contributed to the observed variation in CT_max_ and CT_min_ along the gradient. We also tested whether CT_max_ and CT_min_ exhibit different rates of change given that critical thermal limits (and their plasticity) may have evolved differently in response to different temperature constraints along the gradient. Variation of critical thermal traits was significantly correlated with species’ elevational midpoint, their maximum and minimum elevations, as well as the maximum air temperature and the maximum operative temperature as measured across this gradient. Both thermal limits showed substantial variation, but CT_min_ exhibited relatively faster rates of change than CT_max_, as observed in other taxa. Nonetheless, our findings call for caution in assuming inflexibility of upper thermal limits and underscore the value of collecting additional empirical data on species’ thermal physiology across elevational gradients.

## 1 Introduction

In a rapidly changing world, many species are faced with shrinking habitat and novel climatic conditions. As a result, there has been widespread interest in understanding species responses to past and present climatic variation in order to predict how best to conserve species in future climatic conditions (e.g., Moritz & Agudo, 2000; Sinervo et al., 2010). While much attention has been given to modeling and predicting elevational range shifts in montane organisms, especially in the context of climate change, most predictions about future geographic ranges are based on correlative models that ignore species’ evolutionary history and eco‐physiology (Colwell, Brehm, Cardelús, Gilman, & Longino, 2008; Laurance et al., 2011; VanDerWal et al., 2012). Tropical montane regions are of special concern because they are centers of biodiversity and endemism (Graham et al., 2014). Mountain uplift, climatic fluctuations, and the emergence of new ecological conditions have been hypothesized to promote the diversification of organisms at high elevations (Hoorn et al., 2010; Moritz, Patton, Schneider, & Smith, 2000). As a result, species living at high elevation often exhibit narrowly overlapping (i.e., parapatric) distributions, and are assumed to have greater tolerance to cold (Ghalambor, Huey, Martin, Tewksbury, & Wang, 2006; Janzen, 1967; Navas, 2005). However, empirical data on critical thermal limits of most tropical montane taxa remain unknown. Furthermore, tropical lowland taxa, especially ectotherms, are thought to live near their thermal optimum, so increased temperatures due to changing climates would lead to decreased fitness (Colwell et al., 2008; Huey et al., 2009; Sunday et al., 2014). As with species living at higher elevations, empirical data on species’ critical thermal limits are not available for most tropical lowland taxa.

Several hypotheses have been proposed to explain the potential causes of diversity patterns along elevational gradients (Graham et al., 2014; Hofer, Bersier, & Borcard, 1999; Lomolino, 2001; MacArthur, 1972; McCain & Colwell, 2011; McCain & Grytnes, 2010; Peters et al., 2016; Terborgh, 1970). One of these hypotheses proposes that climatic conditions along the gradient restrict species’ distributions (von Humboldt, 1849; Janzen, 1967). Air temperature is the main environmental factor that predictably decreases with increasing elevation as a result of adiabatic cooling (on average 5.2–6.5°C decrease per 1,000 m elevation; Colwell et al., 2008). Critical thermal maximum (CT_max_) and critical thermal minimum (CT_min_) are two measures that have been used to infer species’ critical thermal limits. Numerous studies have shown that ectotherms exhibit a general trend of decreasing critical thermal limits with elevation (Catenazzi, Lehr, & Vredenburg, 2014; Christian, Nunez, Clos, & Diaz, 1988; Gaston & Chown, 1999; Heatwole, Mercado, & Ortiz, 1965; Navas, 2003). Moreover, it is likely that critical thermal limits change at different rates in response to different temperature constraints along elevational gradients (McCain & Grytnes, 2010). Specifically, CT_max_ is thought to be relatively inflexible across elevation (e.g., Hoffmann, Chown, & Clusella‐Trullas, 2013; Muñoz et al., 2014, 2016), with a narrow upper limit and low plasticity (Gunderson & Stillman, 2015; Sunday, Bates, & Dulvy, 2011).

Although many researchers have examined the relationship between critical thermal limits and the elevational distribution of species living in montane gradients, only a few have combined empirical (CT_max_ and CT_min_) data and accounted for the effect of phylogenetic relatedness among species (Muñoz et al., 2014, 2016; Sheldon, Leaché, & Cruz, 2015). Phylogenetic comparative methods are particularly useful for this purpose because they allow researchers to examine evolutionary transitions in physiological traits and account for statistical nonindependence of interspecific data when studying life history evolution among closely related species (Garland et al. 1992; Harvey & Pagel, 1991; Revell, 2008).

**Figure 1:**
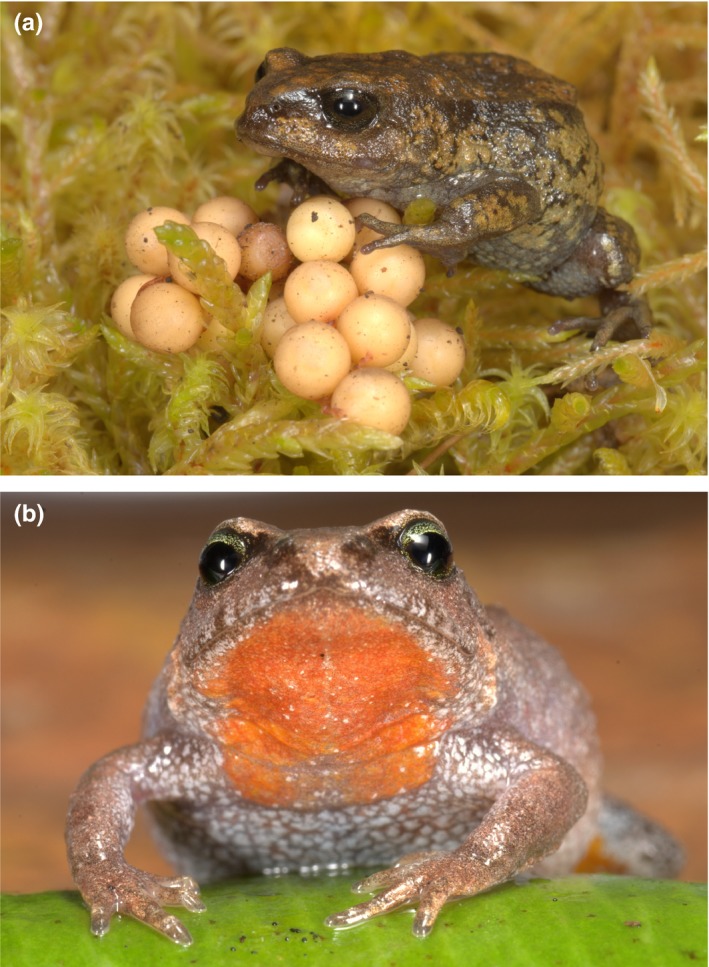
(a) Female *Bryophryne cophites* attending a clutch of direct‐developing embryos at high elevation (above 3200 m a.s.l.). These frogs tolerate near‐freezing temperatures (which they experience during the dry season) as well as moderately high temperatures (which they may experience during sunny days). (b) *Bryophryne hanssaueri* individuals have bright orange coloration ventrally, including the throat. These frogs live under mosses and leaf litter in the high‐elevation cloud forest between 3195 and 3430 m, just below the treeline. Like other *Bryophryne* species, females attend clutches of direct‐developing embryos until they hatch into tiny froglets. Photographs by A. Catenazzi

We investigated the role of physiological divergence among closely related species distributed along an elevational gradient of >3,500 m in southern Peru. Although 80% of Peruvian Andean frogs (ca. 250 species) occur within relatively narrow elevational ranges (Aguilar et al., 2010), little is known about the relationship between their critical thermal limits and their elevational distributions. We focused on 22 species of terrestrial breeding frogs, Craugastoridae, the most diverse amphibian family in the Tropical Andes (Duellman & Lehr, 2009; Hedges, Duellman, & Heinicke, 2008; Padial, Grant, & Frost, 2014). These direct‐developing frogs (Figure 1) are ideal model organisms in which to test hypotheses about divergence across environmental gradients because they have low vagility (resulting in local genetic structure), small body size (a trait that makes them amenable for physiological experiments), and limited geographic and elevational ranges (suggesting strong potential for local adaptation).

Our goal was to examine how CT_max_ and CT_min_ vary in relation to the elevational distribution of species and to test whether life history traits such as body size and body mass, and elevational range midpoint explain differences in CT_max_ and CT_min_ among species. Altogether, we used four metrics relating to elevation (elevational minimum, maximum, midpoint, and range) and two metrics relating to temperature (maximum air temperature and maximum operative temperature) as proxy for thermal environments. We reconstructed a phylogeny to determine the evolutionary relatedness among species and to evaluate the relationship between critical thermal limits and elevation using phylogenetic comparative methods. We tested for phylogenetic signal in all life history traits to infer the role of niche conservatism, which is when related species resemble each other more than expected under a Brownian motion model of trait evolution (Losos, 2008). We also tested whether CT_max_ and CT_min_ are correlated with one another, and determined which life history traits can explain the observed variation in CT_max_ and CT_min_. Furthermore, given that recent studies focusing on thermal niche evolution of terrestrial ectotherms showed that tolerance to cold changes more than tolerance to heat (Araújo et al., 2013; Hoffmann et al., 2013; Muñoz et al., 2014; Sunday et al., 2011), we evaluated whether CT_max_ and CT_min_ exhibited different rates of thermal physiological change.

## 2 Material and methods

### 2.1 Study area

We worked in Manu National Park and its surrounding habitat in southern Peru. Key study sites along the elevational transect included in this study are Acjanaco (13°11′56″S, 71°37′03″W, 3,700 m a.s.l.), Wayqecha Biological Station (13°10′29″S, 71°35′14″W ), San Pedro Cock of the Rock Biological Station (13°03′16″S, 71°32′45″W, 1,400 m a.s.l.), Villa Carmen Biological Station (12°53′44″S, 71°24′14″W, 530 m a.s.l.), and Los Amigos Biological Station (12°34′07″S, 70°05′57″W, 250 m a.s.l.). A general overview of the study sites and local climate was provided by Catenazzi, Lehr, Rodríguez, and Vredenburg (2011) and von May et al. (2009), and Catenazzi, Lehr, and von May (2013) provided an inventory of the herpetofauna in this region.

### 2.2 Field surveys and critical thermal limits

All species surveyed in this study are distributed within the watershed of the Madre de Dios river and along a single montane gradient. Data collected for this study were obtained from multiple surveys conducted along the elevational gradient from Los Amigos Biological Station at 250 m a.s.l. (von May & Donnelly, 2009; von May et al., 2009, 2010) to Tres Cruces at 3,800 m a.s.l. (Catenazzi & Rodriguez, 2001; Catenazzi et al., 2011, 2013, 2014). We measured CT_max_ and CT_min_ in 22 and 14 species, respectively, expanding the taxonomic diversity, number of individuals sampled per species, and elevational coverage of a previous study (Catenazzi et al., 2014). Animals were captured in the field and transported to a field laboratory, where they were kept in individual containers with water ad libitum. All individuals were housed at 16–21°C for 2–3 days prior to measurements. Thus, our measures relate to thermal limits under field conditions, and are likely influenced by both plasticity and adaptation. We used nonlethal experiments to evaluate critical thermal maxima (CT_max_) and minima (CT_min_). CT_max_ and CT_min_ were measured as the point where frogs lost their righting response, defined as the moment when a frog cannot right itself from being placed venter‐up for a period longer than 5 s (Catenazzi et al., 2014; Navas, 1997, 2003). We placed each individual in a plastic cup with a thin layer of water (3–5 mm) and immersed the cups in a water bath. For CT_max_, the bath temperature was progressively increased from 18°C to up to ~35°C at a rate of ~1°C/min by adding warm water. For CT_min_, the temperature was progressively decreased from 18°C to ~0°C by adding ice to the water bath (Christian et al., 1988). We forced animals to a venter‐up position; whenever animals were unable to right themselves for 5 s, we used a quick‐reading thermometer to measure temperature against the body of the frog immersed in the thin layer of water. Given the small size of these frogs, we assumed that this temperature is equivalent to the core temperature of frogs (Navas et al. 2007). The righting response is relevant for considering selection on thermal physiology, because a frog that is unable to display their automatic righting reflex will likely be unable to escape predators. We measured CT_max_ in 768 individuals (22 species) and CT_min_ in 196 individuals (14 of the 22 species). Even though there are fewer data points for CT_min_, our sampling covered the entire gradient for both critical thermal traits.

### 2.3 Laboratory methods

We collected DNA sequence data for two mitochondrial and two nuclear genes in order to determine the phylogenetic relationships among focal species. The mitochondrial genes were a fragment of the 16S rRNA gene and the protein‐coding gene cytochrome c oxidase subunit I (COI). The nuclear protein‐coding genes were the recombination‐activating protein 1 (RAG1) and tyrosinase precursor (Tyr). Extraction, amplification, and sequencing of DNA followed protocols previously used for terrestrial breeding frogs (Hedges et al., 2008; Lehr, Fritzsch, & Müller, 2005). Primers used are listed in Table S1, and we employed the following thermocycling conditions to amplify DNA from each gene using the polymerase chain reaction (PCR). For 16S, we used as follows: 1 cycle of 96°C/3 min; 35 cycles of 95°C/30 s, 55°C/45 s, 72°C/1.5 min; 1 cycle 72°C/7 min. For RAG, we used as follows: 1 cycle of 96°C/2 min; 40 cycles of 94°C/30 s, 52°C/30 s, 72°C/1.5 min; 1 cycle 72°C/7 min. For Tyr, we used as follows: 1 cycle of 94°C/5 min; 40 cycles of 94°C/30 s, 54°C/30 s, 72°C/1 min; 1 cycle 72°C/7 min. We performed the cycle sequencing reactions using BigDye Terminator 3.1 (Applied Biosystems) and ran the purified reaction products on an ABI 3730 Sequence Analyzer (Applied Biosystems). Newly obtained sequences generated in this study were deposited in GenBank (Table S2).

### 2.4 Phylogenetic analysis

We used Geneious R6, version 6.1.8 (Biomatters 2013; http://www.geneious.com/) to align the sequences using the built‐in multiple alignment program. For 16S, we visualized the alignment, trimmed the ends, and removed the highly variable noncoding loop regions (to avoid alignment artifacts). Prior to conducting phylogenetic analysis, we used PartitionFinder, version 1.1.1 (Lanfear, Calcott, Ho, & Guindon, 2012) to select the appropriate models of nucleotide evolution. We used the Bayesian information criterion (BIC) to determine the best partitioning scheme and substitution model for each gene. The best fitting substitution model for 16S was GTR+I+G. The best partitioning scheme for COI and both nuclear genes included specific sets according to codon positions. For COI, the best partitioning scheme included three sets of sites (substitution models in parentheses): the first set with 1st codon position (K80 + G), the second set with 2nd codon position (HKY), and the third set with the 3rd codon position (TrN+G). For RAG, the best partitioning scheme included two sets of sites: the first set with 1st and 2nd codon positions together (HKY+I) and the second set with only the 3rd codon position (K80 + G). Likewise, for Tyr, the best partitioning scheme included two sets of sites: the first set with 1st and 2nd codon positions together (K80 + I) and the second set with only the 3rd codon position (K80 + G). We inferred nuclear haplotypes from genotype data using PHASE version 2.1 (Stephens & Scheet, 2005; Stephens, Smith, & Donnelly, 2001) and processed the input and output files with SEQPHASE (Flot, 2010).

We used a multispecies coalescent approach implemented in *BEAST v1.6.2 (Drummond & Rambaut, 2007) to infer a Bayesian multilocus timetree of the 22 focal taxa. The primary goal of the analysis was to obtain an ultrametric tree to be used for phylogenetic comparative analyses. Our analyses only depend on the relative branch lengths of the tree, but we preferred to illustrate our tree in rough units of time. Therefore, we used an uncorrelated relaxed molecular clock with the rate of nucleotide substitution for 16S was set at 1% per million years. However, we note that the dates associated with the tree should only be viewed as very approximate and that there can be multiple sources of error when calibrating phylogenies (Arbogast, Edwards, Wakeley, Beerli, & Slowinski, 2002). The analysis in *BEAST included two independent runs, each with 100 million generations and sampled every 10,000 generations. Following the completion of the analysis, we used Tracer v1.5 (Rambaut & Drummond, 2007) to examine effective sample sizes, verify convergence of the runs, and to ensure the runs had reached stationarity. Observed effective sample sizes were sufficient for most parameters (ESS > 200) except for substitution rates for a few partitions. We discarded the first 10% of samples from each run as burn‐in. Subsequently, we used LogCombiner v1.6.2 to merge all remaining trees from both runs and used TreeAnnotator v1.6.2 (Drummond & Rambaut, 2007) to summarize trees and obtain a maximum clade credibility tree (available at the Dryad Digital Repository: doi:10.5061/dryad.84bp7). We visualized the MCC tree and the associated node support values in FigTree (http://tree.bio.ed.ac.uk/software/figtree/).

### 2.5 Phylogenetic signal

For a given quantitative trait, phylogenetic signal is present when related species tend to resemble one another (Blomberg, Garland, & Ives, 2003; Harvey & Pagel, 1991). We tested for phylogenetic signal by calculating the *K* (Blomberg et al., 2003) and λ statistics (Pagel, 1999) in the R package ‘phytools’ (Revell, 2010a, 2010b). Both methods are commonly used to account for nonindependence of interspecific data resulting from shared ancestry (Ashton, 2004; Corl, Davis, Kuchta, Comendant, & Sinervo, 2010; Revell, 2008). For *K*, values smaller than 1 indicate that related species are less similar than expected under a Brownian motion model of trait evolution while values greater than 1 indicate that related species resemble each other more than expected under a Brownian motion model of trait evolution (Blomberg et al., 2003). The value of λ typically ranges between 0, indicating no phylogenetic signal, and 1, indicating strong phylogenetic signal (i.e., when related species resemble each other more than expected under a Brownian motion model of evolution) (Pagel, 1999). For CT_max_ and CT_min_, phylogenetic signal tests were done both considering and not considering intraspecific measurement error in either CT_max_ or CT_min_ values. Given that considering measurement error did not affect the results, only results from tests with no measurement error are included in the Results section.

### 2.6 Rates of thermal physiological change

Prior to comparing the rates of physiological change for CT_max_ and CT_min_, we searched for a model of evolution that best explains the variation in the observed data. We used the fitContinuous function in GEIGER (Harmon, Weir, Brock, Glor, & Challenger, 2008) to fit three models of evolution: Brownian motion (BM), Ornstein–Uhlenbeck (OU), and early burst (EB). The Brownian motion model assumes a constant rate of change, such that the differences between species will (on average) be proportional to the time since their divergence. The Ornstein–Uhlenbeck model assumes a stationary distribution, such that the differences between species will not necessarily relate to their time since divergence. Finally, the early burst model assumes an exponential decline in rates through time. This means that species with recent divergence times will be very similar, while species with deeper divergences will be relatively independent of one another. After determining the best fitting model of evolution for each trait, we used the R package “APE” (Paradis, Claude, & Strimmer, 2004) and code developed by Adams (2013) to estimate the rates of change.

### 2.7 Correlates of CT_max_ and CT_min_

We explored the relationship between critical thermal traits and other life history characteristics (body size and body mass) as well as four metrics relating to elevation—minimum, maximum, midpoint, and range collected from 22 species of Craugastoridae frogs. We also considered maximum air temperatures (*T*_*a*_) and maximum operative temperatures (*T*_*e*_), both of which were previously estimated for the same montane gradient (Catenazzi et al., 2014). The *T*_*a*_ data were inferred by regressing daily average temperatures vs. elevation from four weather stations operated by Peru's national weather service (SENAMHI = Servicio Nacional de Meteorología e Hidrología del Perú) from 520 to 3,485 m a.s.l. The *T*_*e*_ data were inferred from field measurements taken with 21 iButtons (Maxim Integrated Products, Sunnyvale, California, USA) placed in forest microhabitats used by frogs at five sites between 1,525 and 3,500 m. For two species that are primarily distributed in the Andean grassland (*Bryophryne cophites* and *Psychrophrynella usurpator*), *T*_*e*_ data were inferred from measurements taken with 12 iButtons placed in this microhabitat from 2,800 to 3,450 m. Furthermore, as in Catenazzi et al. (2014), we calculated operative warming tolerances (OWTs) by subtracting the average maximum *T*_*e*_ from CT_max_. We also considered the thermal breadth, defined as the difference between CT_max_ and CT_min_. We examined a correlogram displaying the relationships between pairs of variables (Fig. S1) to determine which predictor variables were highly correlated with each other. We used the R package “phylolm” (Ho & Anné, 2014a, 2014b) to fit phylogenetic generalized linear regression models. This package implements a phylogenetic regression under various models for the residual error, including Brownian motion (BM) and Ornstein–Uhlenbeck (OU; these models were implemented with a constant selection strength α and variance rate σ^2^). We used the AIC value to identify the model that best explains the variation of observed data (Ho & Anné, 2014b).

## 3 Results

### 3.1 Phylogenetic relatedness and elevational distribution

**Figure 2:**
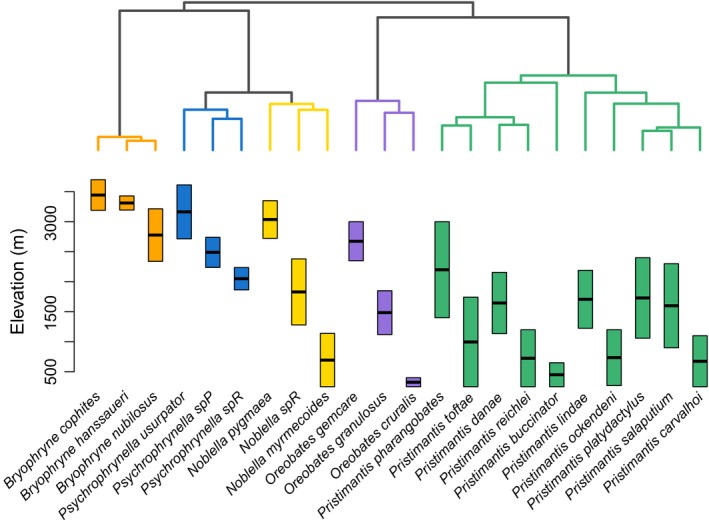
Elevational divergence in terrestrial breeding frogs along a tropical montane gradient. Species tree (obtained with *BEAST) depicting the relationship among the 22 species included in this study (top) and their elevational distribution along the study transect (bottom). The elevational midpoint is denoted by a black bar. Species are color‐coded according to genus

We recovered a well‐supported phylogenetic tree (Figure 2 and Fig. S2; node support values shown in Fig. S2) that was generally congruent with previous trees (Padial et al., 2014). Seventeen of 21 nodes had Bayesian posterior probabilities greater than 0.95 (Fig. S2). We mapped elevational data on to the species tree obtained with *BEAST to visually assess the patterns of elevational distribution and phylogenetic relatedness (Figure 2).

We observed that closely related, congeneric species exhibit generally parapatric distributions with respect to elevation; an exception to this pattern was seen in some species of *Pristimantis* (e.g., *P. platydactylus* and *P. salaputium*) that exhibit broader elevational overlap (Figure 2). A congruent and similarly well‐supported phylogeny was obtained with a concatenated partitioned dataset analyzed with MrBayes (Ronquist & Huelsenbeck, 2003; see Supporting Information and Fig. S3).

### 3.2 Critical thermal traits

**Figure 3:**
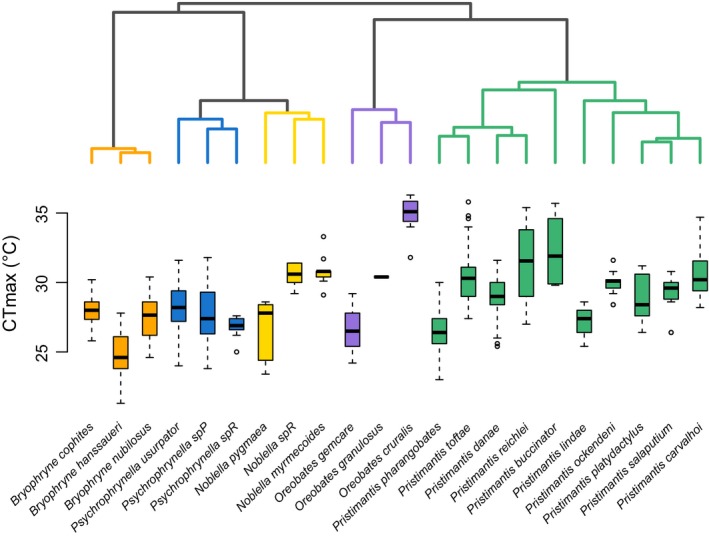
Divergence in CT_max_ in terrestrial breeding frogs along a tropical montane gradient. Species tree (obtained with *BEAST) depicting the relationship among the 22 species included in this study (top) and box plots depicting their CT_max_ values (bottom). The box plots show the median (black bar), interquartile range (box), and 1.5 times the interquartile range (bars); circles represent outliers. Species are color‐coded according to genus

We observed substantial differences in CT_max_ values (from 24.8°C to 34.8°C) among both closely and distantly related species (Figure 3; Table S3). In five cases, close relatives had nonoverlapping CT_max_ values and nonoverlapping elevational distributions. The highest CT_max_ was found in *Oreobates cruralis*, an exclusively lowland species, and the lowest CT_max_ was found in *Bryophryne hanssaueri*, a species distributed in highland forests just below the treeline. CT_min_ also varied substantially across the gradient (from 1.6°C to 15.2°C; Table S3). In three cases, close relatives exhibited nonoverlapping CT_min_ values (Fig. S4).

### 3.3 Phylogenetic signal

No phylogenetic signal was detected for CT_max_, in tests both considering and not considering intraspecific measurement error in CT_max_ values (Table 1; only results from tests with no measurement error are shown). This result infers that, for CT_max_, closely related species are less similar than expected from a Brownian motion model of evolution along the tree. Likewise, no phylogenetic signal was detected for CT_min_, based on a test using the reduced dataset (14 species). In contrast, a strong phylogenetic signal was detected for both SVL and body mass, and a moderate phylogenetic signal for minimum elevation, maximum elevation, elevational midpoint, and elevational range (Table 1). The only discrepancy observed between the two phylogenetic signal statistics was observed for maximum elevation (λ nonsignificant) and elevational range (λ marginally nonsignificant).

**Table 1:** Results from the tests for phylogenetic signal based on two statistics, *K* and λ. Log likelihood values included correspond to the λ estimates. Phylogenetic signal tests were done with the full dataset (22 species) for all traits except for CT_min_. Phylogenetic signal tests were conducted for CT_min_ and repeated for CT_max_ with the reduced dataset (14 species)

Trait	*K*	*p*‐value _(*K*)_	λ	*p*‐value _(λ)_	lnL
Analyses with full dataset (22 species)					
CT_max_	0.3955	.1572	0.0626	.8202	−49.22
SVL	0.9548	**.0010**	1.0352	**.0003**	−64.71
Mass	0.7589	**.0030**	1.0560	**.0031**	−24.61
Minimum elevation	0.7011	**.0020**	0.7291	**.0055**	−179.52
Maximum elevation	0.5233	**.0140**	0.3854	.1559	−181.17
Elevational midpoint	0.6115	**.0060**	0.5903	**.0307**	−180.03
Elevational range	0.4944	**.0280**	0.4999	.0635	−160.54
Analyses with reduced dataset (14 species)					
CT_min_	0.7019	.0631	1.1339	.0681	−35.68
CT_max_	0.5279	.2302	0.0001	1.000	−30.25

Bold indicates significant phylogenetic signal.

### 3.4 Rates of thermal physiological change

Results of fitting tests for the three models of trait evolution showed that BM was the best model for both CT_max_ and CT_min_ (Table S4). The method used for estimating the rates of evolution (Adams, 2013) assumes a constant rate of change (BM), and we performed this test assuming BM for both traits and using the reduced dataset (14 species). We found that CT_max_ exhibits a slower rate of change than CT_min_ (σ^2 ^= 0.686 and σ^2^ = 1.353, respectively; likelihood ratio test, LRT = 4.443, AICc = 128.319, *p *=* *.035).

### 3.5 Correlates of CT_max_ and CT_min_

**Figure 4:**
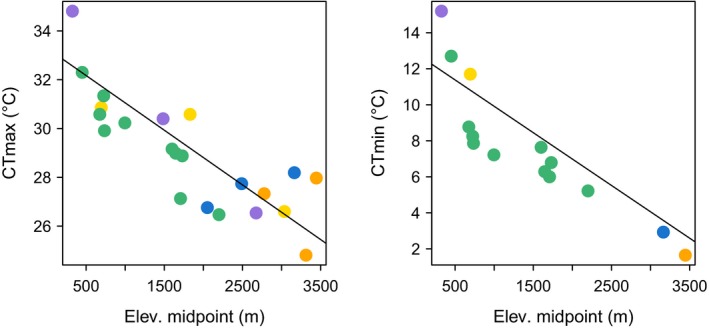
Correlation between CT_max_ and elevational midpoint (left) and between CT_min_ and elevational midpoint (right). Species are color‐coded according to genus (see Figures 2 and 3). The slopes of the regression lines reflect the phylogenetic corrections in each model

Phylogenetic linear regression models indicated that CT_max_ and CT_min_ were significantly correlated with all proxies of thermal environment—minimum elevation, maximum elevation, elevational midpoint, maximum air temperature, and maximum operative temperature (Table 2, Table 3). In all cases, increasing elevation led to decreasing CT_max_ and CT_min_ (Figure 4, Table 2, Table 3). Body size, body mass, and elevational range did not explain the variation in CT_max_ and CT_min_ (Table 2, Table 3). Models with two or more variables did not provide a better fit compared to univariate models (i.e., AIC values of models with two or more variables were greater than AIC values of univariate models; Table S5). Further, CT_max_ and CT_min_ were significantly correlated with one another (AIC = 53.46, log likelihood = −23.73, *p *=* *.0003; reduced dataset of 14 species).

**Table 2:** Results from phylogenetic generalized linear regression models for CT_max_, fitted assuming the Brownian motion (BM) model of evolution. Model fitting was done with the full dataset (22 species). Similar results were obtained with the Ornstein–Uhlenbeck (OU) model (results not shown). *T*_a_ = maximum air temperature; *T*_e_ = maximum operative temperature

Model	Evol. model	Coefficient	*p*‐value	AIC	lnL
CT_max_ ~ min_elev	BM	−0.0023	**<.001**	90.02	−42.01
CT_max_ ~ max_elev	BM	−0.0020	**<.001**	93.24	−43.62
CT_max_ ~ elev_midpoint	BM	−0.0022	**<.001**	90.21	−42.10
CT_max_ ~ elev_range	BM	−0.0002	.881	110.27	−52.13
CT_max_ ~ *T*_*a*_	BM	0.3542	**<.001**	89.09	−41.55
CT_max_ ~ *T*_*e*_	BM	0.4782	**<.001**	69.28	−31.64
CT_max_ ~ svl	BM	−0.1844	.136	107.78	−50.89
CT_max_ ~ mass	BM	−1.191	.115	107.49	−50.75

Bold indicates significant effect.

**Table 3:** Results from phylogenetic generalized linear regression models for CT_min_, fitted assuming the Brownian motion (BM) model of evolution. Model fitting was done with the reduced dataset (14 species). Similar results were obtained with the Ornstein–Uhlenbeck (OU) model (results not shown). *T*_*a*_ = maximum air temperature; *T*_*e*_ = maximum operative temperature

Model	Evol. model	Coefficient	*p*‐value	AIC	lnL
CT_min_ ~ min_elev	BM	−0.0031	**<.001**	58.99	−26.50
CT_min_ ~ max_elev	BM	−0.0026	**<.001**	56.39	−25.20
CT_min_ ~ elev_midpoint	BM	−0.0029	**<.001**	56.14	−25.07
CT_min_ ~ elev_range	BM	0.0041	.081	75.17	−34.59
CT_min_ ~ *T*_*a*_	BM	0.4728	**<.001**	51.74	−22.87
CT_min_ ~ *T*_*e*_	BM	0.5998	**<.001**	58.26	−26.13
CT_min_ ~ svl	BM	−0.2730	.138	76.19	−35.10
CT_min_ ~ mass	BM	−1.2024	.358	77.83	−35.92

Bold indicates significant effect.

**Figure 5:**
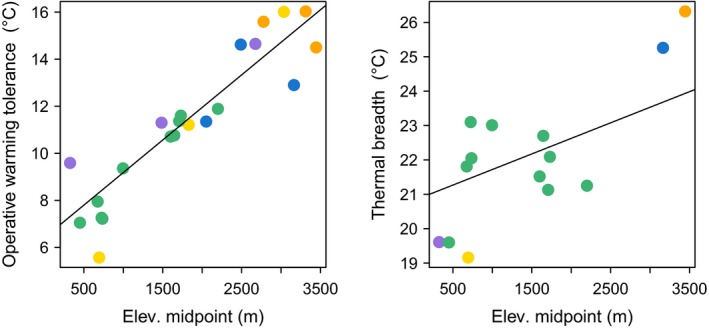
Correlation between operative warming tolerance and elevational midpoint (left) and correlation between thermal breadth (= CT_max_ − CT_min_) and elevational midpoint (right). Species are color‐coded according to genus, and the regression lines reflect the phylogenetic correction

Our data also showed that operative warming tolerance (OWT) increased with elevation (AIC = 86.90, log likelihood = −40.45, *p *<* *.001; Figure 5). Therefore, the distance between CT_max_ and maximum operative temperature (T_e_) of high‐elevation species is greater than that of species distributed at lower elevations. We also observed a consequent increase in thermal breadth (= CT_max_ − CTmin) at higher elevations, although this relationship was marginally nonsignificant (AIC = 59.87, log likelihood = −26.94, *p *<* *.0831; Figure 5).

## 4 Discussion

Our findings suggest that thermal physiology is relevant in determining where species live, and provide further evidence that local adjustment to the thermal environment, whether by plasticity or adaptation, is an important process in tropical mountains (Cadena et al., 2012). Overall, critical thermal limits decreased with elevation as well as with decreasing air (*T*_*a*_) and operative (*T*_*e*_) temperatures, a pattern exhibited by other terrestrial ectotherms living along montane gradients (Christian et al., 1988; Gaston & Chown, 1999; Muñoz et al., 2014; Navas, 2003).

Importantly, the high variability observed in both CT_max_ and CT_min_ among closely related species (Figure 3 and Fig. S4) supports the idea that thermal traits in ectotherms can adjust through evolutionary time. In contrast to studies focusing on thermal physiology across distantly related taxa (i.e., different families) and/or larger geographic scales (e.g., Araújo et al., 2013; Kellermann, Loeschcke, et al., 2012; Kellermann, Overgaard, et al., 2012; Sunday et al., 2014), we investigated species within a single family distributed along a steep elevational gradient. We believe this approach can be used to refine predictions and to test further hypotheses regarding physiological divergence among montane taxa, especially if such studies incorporate knowledge of phylogenetic relatedness among species. Synthesizing this information is essential for understanding historical patterns and processes determining species’ elevational distributions and for predicting species’ responses to climate change (Moritz & Agudo, 2000).

Our tests of phylogenetic signal focusing on CT_max_ indicated that closely related species are less similar than expected under a Brownian motion model of evolution, supporting the idea that even upper thermal limits can change rapidly in this diverse amphibian clade. This finding, along with those from Neotropical plethodontid salamanders (Kozak & Wiens, 2007), suggests that niche divergence in tolerance to heat may be common among montane amphibians (e.g., Navas, 1997, 2003). Our tests of phylogenetic signal focusing on CT_min_ based on a reduced dataset (14 species) also suggested that closely related species tend to differ in their tolerance to cold. The reduced dataset for CT_min_ spans the full elevational range, but had few species distributed at high elevation (e.g., only one species of *Bryophryne* and only one *Psychrophrynella*), so an expanded dataset is required to examine this pattern more thoroughly. Given that CT_max_ and CT_min_ are significantly correlated with one another, and that each of these traits is significantly correlated with elevational midpoint, maximum elevation, and minimum elevation, we predict that an expanded dataset for CT_min_ will support the hypothesis that tolerance to cold has changed rapidly in this clade. Given that the Andes have experienced multiple uplift events since the Miocene (Hoorn et al., 2010), the emergence of colder environments along the montane gradient might have promoted rapid divergence in species’ thermal physiological traits. These observations for amphibians contrast with experimental studies of *Drosophila*, where there appears to be strong phylogenetic constraint on both cold and heat tolerance (Kellermann, Loeschcke, et al., 2012; Kellermann, Overgaard, et al., 2012).

Nevertheless, observing strong correlations does not necessarily imply that either the lower or upper bound of the elevational range of montane frog species is constrained by their critical thermal limits (Catenazzi, 2011; Navas, 1997). In addition to species’ thermal physiology, factors such as availability of breeding sites, competition, predation, and other biotic interactions may play an important role in restricting species’ elevational distribution (Hutchinson, 1957; Jankowski, et al., 2013; Terborgh & Weske, 1975; Wake & Lynch, 1976). Likewise, other climatic factors such as rainfall, relative humidity, and availability of microrefugia in the dry season may also play a role in determining the upper and lower elevational range limits in (Hutchinson, 1957; Jankowski, et al., 2013; Terborgh & Weske, 1975; Wake & Lynch, 1976).

Our finding that CT_min_ has faster rates of change than CT_max_ is consistent with results from phylogenetic comparisons of sets of related lizards distributed across elevational gradients in the tropics (e.g., Muñoz et al., 2014, 2016). Nevertheless, differences in species distributions and in species’ thermoregulation strategies between frogs and lizards might reflect contrasting patterns of physiological evolution. While lizards tend to occur in warm places where they can actively thermoregulate, frogs occur in greater numbers in cold environments and most species are considered to be thermoconformers (Navas, 2003)—with the notable exceptions of some high‐elevation frog species that thermoregulate opportunistically (Navas, 1997). For example, the mountaintop at our study site (~3,500 m elevation) is inhabited by eight frog species of three families, but only one lizard species. Therefore, the selective pressures on thermal limits are likely to differ largely between frogs and lizards.

Several studies focusing on terrestrial ectotherms have suggested that plasticity may not play an important role in shaping interspecific variation in critical thermal limits. For example, a recent meta‐analysis by Gunderson and Stillman (2015) found that terrestrial ectotherms exhibit low acclimation potential (i.e., low plasticity) for heat resistance. However, this hypothesis requires further testing and the group of tropical frogs studied here represents a suitable study system to examine the contribution of plasticity vs. genetic effects. Future studies should examine variation in the acclimation potential of montane and high‐elevation tropical frogs, complementing previous studies that found no such capacity, or very low acclimation potential, in frogs (Brattstrom, 1968; Christian et al., 1988; Gunderson & Stillman, 2015).

Our findings do not support a broad assumption of niche conservatism in research aimed at examining species’ responses to environmental change. Many researchers have used species distribution modeling approaches to predict whether species will experience range shifts or extinction in the face of climate warming (Chen, Hill, Ohlemüller, Roy, & Thomas, 2011; Laurance et al., 2011; VanDerWal et al., 2012). The assumption underlying many of these studies is that climatic niches have not changed along the history of species, both within and among closely related species (Wiens et al., 2010). However, our results call for caution in assuming inflexibility of thermal limits, especially CT_max_, in montane anurans, and underscore the value of collecting additional empirical data on species’ thermal physiology (Perez, Stroud, & Feeley, 2016). It is worth noting that while our results suggest that thermal limits may change rapidly on the time scale of the formation of new species, it is still an open question about whether thermal physiology will be able to keep pace with future global climate change that may be more rapid than in the recent past (Gunderson & Stillman, 2015). Our data on operative warming tolerance (Figure 5) support the idea that tropical lowland species might be more sensitive to increased temperatures than high‐elevation species, because they live at ambient conditions that are closer to their critical thermal limits (Colwell et al., 2008; Huey et al., 2009; Sunday et al., 2014). In turn, tropical amphibians living at high elevation might be more buffered from increased temperatures, as their CT_max_ values are farther away from the maximum temperatures that they regularly experience in the wild (Catenazzi et al., 2014). More studies on populations/species that have recently diverged along montane gradients are needed to help estimate maximal rates of change of thermal limits.

## Conflict of interest

The authors have declared that no conflict of interests exist.
