# Supplementary material for: Divergence of thermal physiological traits in terrestrial breeding frogs along a tropical elevational gradient
Source: Ecol Evol. 2017 Apr 6;7(9):3257–67. doi: 10.1002/ece3.2929 (PMC5415528; doi:10.1002/ece3.2929)
Supplement: Supplementary file 1 [file ECE3-7-3257-s001.docx]

**Supplementary materials for article**

**Divergence of thermal physiological traits in terrestrial breeding frogs along a tropical elevational gradient**

Rudolf von May, Alessandro Catenazzi, Ammon Corl, Roy Santa-Cruz, Ana Carolina Carnaval, Craig Moritz

**Methods**

**Bayesian Inference using partitioned dataset.** We employed a Bayesian approach using MrBayes, version 3.2.0 (Ronquist & Huelsenbeck 2003) to infer a molecular phylogeny. Our analysis included 22 terminal taxa and a 2368-bp concatenated partitioned dataset. This dataset included the mitochondrial 16S and COI fragments and both the RAG and Tyr nuclear genes. We performed an MCMC Bayesian analysis that consisted of two simultaneous runs of 10 million generations, and we set the sampling rate to be once every 1000 generations. Each run had three heated chains and one “cold” chain, and the burn-in was set to discard the first 25% samples from the cold chain. At the end of the run, the average standard deviation of split frequencies was 0.007603. Following the completion of the analysis, we used Tracer v1.5 (Rambaut & Drummond 2007) to verify convergence. Subsequently, we used FigTree (http://tree.bio.ed.ac.uk/software/figtree/) to visualize the majority-rule consensus tree and the posterior probability values to assess node support.

**References**

Rambaut, A. and A.J. Drummond. 2007. Tracer. Version 1.5. Available at: http://tree.bio.ed.ac.uk/ software/tracer/.

Ronquist, F. and Huelsenbeck, J.P. 2003. MrBayes 3: Bayesian phylogenetic inference under mixed models. Bioinformatics 19: 1572–1574.

**Supplementary Figures**

**Supplementary** **Figure S1.** Correlogram displaying the correlation between pairs of variables, with Pearson values (font size scaled to value) on the lower half of the matrix. Abbreviations for each variable are included along the diagonal (ctmax = critical thermal maximum; ctmin = critical thermal minimum; svl = snout-vent-length; mass = body mass; T_a_ = maximum air temperatures; T_e_ = maximum operative temperatures; minElev = minimum elevation; maxElev = maximum elevation; midpoint = elevational midpoint; rangeEl = elevational range).

**Supplementary** **Figure S2.** Maximum Clade Credibility tree for 22 species included in this study, based on a multilocus species tree obtained with *BEAST. The node values include Bayesian posterior probabilities and the bars indicate 95% High Posterior Density (HPD) intervals.

**Supplementary** **Figure S3.** Bayesian maximum clade-credibility tree for 22 species included in this study based on a 2368-bp concatenated partitioned dataset (16S, COI, RAG1, Tyr) analyzed in MrBayes (posterior probabilities are indicated at each node). Species are color-coded according to genus (see also Figure 2 in manuscript).

**Supplementary** **Figure S4.** Species tree depicting the relationship among the 14 species with CT_min_ data (top) and box plots depicting their CT_min_ values (bottom). The black bars indicate the median, the boxes indicate the interquartile range, and bars indicate the 1.5 times inter-quartile range; circles represent outliers. Box plots are color-coded according to genus as in Figures 2 and 3.

**Supplementary Tables**

**Supplementary** **Table S1.** Primers used in this study.

| **Locus** | **Primer** |  | **Sequence (5'-3')** | **Reference** |
| --- | --- | --- | --- | --- |
| 16S  COI  COI  Tyr  RAG-1 | 16Sar  16Sbr  dgLCO1490  dgHCO2198  Chmf4  Chmr4  Tyr1C  Tyr1G  R182  R270 | F  R  F  R  F  R  F  R  F  R | CGCCTGTTTATCAAAAACAT  CCGGTCTGAACTCAGATCACGT  GGTCAACAAATCATAAAGAYATYGG  TAAACTTCAGGGT GACCAAARAAYCA  TYTCWACWAAYCAYAAAGAYATCGG  ACYTCRGGRTGRCCRAARAATCA  GGCAGAGGAWCRTGCCAAGATGT  TGCTGGGCRTCTCTCCARTCCCA  GCCATAACTGCTGGAGCATYAT  AGYAGATGTTGCCTGGGTCTTC | Palumbi et al. (1991)  Palumbi et al. (1991)  Meyer et al. (2005)  Meyer et al. (2005)  Che et al. (2012)  Che et al. (2012)  Bossuyt and Milinkovitch (2000)  Heinicke et al. (2007)  Heinicke et al. (2007) |

**Supplementary** **Table S2.** Voucher numbers and GenBank accession numbers for the taxa and genes sampled in this study.

| **Taxon** | **Voucher_Nbr** | **16S** | **COI** | **RAG1** | **Tyr** |
| --- | --- | --- | --- | --- | --- |
| *Bryophryne cophites* | AC270_07 | KY652641 | KY672976 | KY672961 | KY681062 |
| *Bryophryne hanssaueri* | MUSM 27567 | KY652642 | KY672977 | KY681084 | KY681063 |
| *Bryophryne nubilosus* | MUSM 27882 | KY652643 | KY672978 | KY681085 | KY681064 |
| *Noblella myrmecoides* | RvM3_12 | KY652644 | NA | KY672962 | KY681065 |
| *Noblella pygmaea* | MUSM 24536 | KY652645 | KY672979 | KY681086 | KY681066 |
| *Noblella* sp. “Rocotal” | MUSM 27582 | KY652646 | KY672980 | KY681087 | KY681067 |
| *Oreobates cruralis* | MUSM 33248 | KY652647 | NA | KY672963 | KY681068 |
| *Oreobates gemcare* | MUSM 27903 | KY652648 | KY672981 | KY672964 | KY681069 |
| *Oreobates granulosus* | AC94_07 | KY652649 | KY672982 | KY672965 | KY681070 |
| *Pristimantis buccinator* | MUSM 33269 | KY652650 | NA | KY672966 | KY681071 |
| *Pristimantis carvalhoi* | CORBIDI 16294 | KY652651 | KY672983 | KY672967 | KY681072 |
| *Pristimantis danae* | MVZ 272358 | KY652652 | KY672984 | KY672968 | KY681073 |
| *Pristimantis lindae* | MUSM 27902 | KY652653 | KY672985 | KY672969 | KY681074 |
| *Pristimantis ockendeni* | RvM5_12 | KY652654 | KY672986 | KY672970 | KY681075 |
| *Pristimantis pharangobates* | MVZ 272360 | KY652655 | KY672987 | KY681088 | KY681076 |
| *Pristimantis platydactylus* | MVZ 272359 | KY652656 | KY672988 | KY672971 | KY681077 |
| *Pristimantis reichlei* | CORBIDI 16219 | KY652657 | KY672989 | KY672972 | KY681078 |
| *Pristimantis salaputium* | MUSM 27916 | KY652658 | KY672990 | KY672973 | KY681079 |
| *Pristimantis toftae* | AC107_07 | KY652659 | KY672991 | KY672974 | KY681080 |
| *Psychrophrynella* sp. “P” | AC116_09 | KY652660 | KY672992 | KY681089 | KY681081 |
| *Psychrophrynella* sp. “R” | AC148_07 | KY652661 | KY672993 | KY681090 | KY681082 |
| *Psychrophrynella usurpator* | AC186_09 | KY652662 | KY672994 | KY672975 | KY681083 |

**Supplementary** **Table S3.** Summary data for all species included in this study: critical thermal maximum (CT_max_), snout-vent-length (SVL), body mass (Mass), minimum elevation (minElev), maximum elevation (maxElev), elevational midpoint (midpoint), elevational range (range elev), and critical thermal minimum (CT_min_). Units are given in parentheses; mean and standard error of the mean (SE) are given for CT_max_ and CT_min_. The elevational data are based on long-term research at the study area.

| **Species** | **CT_max_ ± SE** | **SVL** | **mass** | **minElev** | **maxElev** | **midpoint** | **range** | **CT_min_ ± SE** |
| --- | --- | --- | --- | --- | --- | --- | --- | --- |
|  | (°C) | (mm) | (g) | (m) | (m) | (m) | (m) | (°C) |
| *Bryophryne cophites* | 28.0 ± 0.1 | 23.5 | 1.2 | 3190 | 3700 | 3445 | 510 | 1.6 ± 0.1 |
| *Bryophryne hanssaueri* | 24.8 ± 0.3 | 20.5 | 0.9 | 3195 | 3430 | 3313 | 235 | — |
| *Bryophryne nubilosus* | 27.3 ± 0.3 | 19.0 | 0.7 | 2340 | 3215 | 2778 | 875 | — |
| *Noblella myrmecoides* | 30.9 ± 0.4 | 9.3 | 0.1 | 250 | 1138 | 694 | 888 | 11.7 ± 0.4 |
| *Noblella pygmaea* | 26.6 ± 0.6 | 12.4 | 0.2 | 2723 | 3350 | 3037 | 627 | — |
| *Noblella* sp. R | 30.6 ± 0.3 | 12.1 | 0.2 | 1280 | 2380 | 1830 | 1100 | — |
| *Oreobates cruralis* | 34.8 ± 0.6 | 23.7 | 1.2 | 250 | 400 | 325 | 150 | 15.2 ± 0.0 |
| *Oreobates gemcare* | 26.5 ± 0.2 | 32.1 | 3.4 | 2350 | 3000 | 2675 | 650 | — |
| *Oreobates granulosus* | 30.4 ± 0.0 | 33.2 | 3.0 | 1120 | 1850 | 1485 | 730 | — |
| *Pristimantis buccinator* | 32.3 ± 1.0 | 22.1 | 0.5 | 250 | 650 | 450 | 400 | 12.7 ± 0.9 |
| *Pristimantis carvalhoi* | 30.6 ± 0.6 | 15.1 | 0.4 | 250 | 1100 | 675 | 850 | 8.8 ± 0.2 |
| *Pristimantis danae* | 29.0 ± 0.1 | 28.2 | 2.0 | 1135 | 2156 | 1646 | 1021 | 6.3 ± 0.1 |
| *Pristimantis lindae* | 27.1 ± 0.9 | 31.1 | 2.5 | 1225 | 2190 | 1708 | 965 | 6.0 ± 0.0 |
| *Pristimantis ockendeni* | 29.9±0.2 | 21.0 | 0.8 | 270 | 1200 | 735 | 930 | 7.9 ± 0.2 |
| *Pristimantis pharangobates* | 26.5 ± 0.2 | 23.9 | 1.2 | 1400 | 3000 | 2200 | 1600 | 5.2 ± 0.1 |
| *Pristimantis platydactylus* | 28.9 ± 0.4 | 18.3 | 0.9 | 1060 | 2400 | 1730 | 1340 | 6.8 ± 0.2 |
| *Pristimantis reichlei* | 31.3 ± 0.4 | 24.5 | 2.3 | 250 | 1200 | 725 | 950 | 8.2 ± 0.2 |
| *Pristimantis salaputium* | 29.2 ± 0.5 | 15.8 | 0.5 | 900 | 2300 | 1600 | 1400 | 7.6 ± 0.2 |
| *Pristimantis toftae* | 30.2 ± 0.3 | 21.4 | 0.8 | 250 | 1742 | 996 | 1492 | 7.2 ± 0.3 |
| *Psychrophrynella* sp. P | 27.7 ± 0.3 | 18.1 | 0.6 | 2240 | 2740 | 2490 | 500 | — |
| *Psychrophrynella* sp. R | 26.8 ± 0.2 | 16.3 | 0.5 | 1865 | 2236 | 2051 | 371 | — |
| *Psychrophrynella usurpator* | 28.2 ± 0.1 | 20.5 | 0.9 | 2715 | 3613 | 3164 | 898 | 2.9 ± 0.1 |

**Supplementary** **Table S4.** Summary table comparing the fit of three models of evolution tested for CT_max_ and CT_min_ data. Likelihood estimates (lnL) and corrected Akaike Information Criterion (AIC_C_) values for tests considering the reduced dataset (i.e., 14 species with both CT_max_ and CT_min_ data) are provided; AIC_C_ values in bold indicate the best-supported model.

|  | **CT_max_** (reduced) | | **CT_min_** (reduced) | |
| --- | --- | --- | --- | --- |
| Model | lnL | AICc | lnL | AICc |
| Brownian Motion | -31.672 | **68.434** | -36.431 | **77.953** |
| Ornstein-Uhlenbeck | -30.249 | 68.898 | -36.431 | 81.262 |
| Early Burst | -31.672 | 71.744 | -36.396 | 81.193 |

**Supplementary** **Table S5.** Results from phylogenetic generalized linear regression models for CT_max_, fitted assuming the Brownian Motion (BM) model of evolution. Model fitting was done with the full dataset (22 species). AIC values of models with two variables were greater than AIC values of univariate models (See Table 2). T_a_ = maximum air temperature; T_e_ = maximum operative temperature.

| **Model** | **Estimate** | **Coefficient** | **P-value** | **AIC** | **lnL** |
| --- | --- | --- | --- | --- | --- |
| CT_max_ ~ svl + elev_midpoint |  |  |  | 92.09 | -42.04 |
|  | svl | 0.0297 | 0.752 |  |  |
|  | elev_midpoint | -0.0023 | **<0.001** |  |  |
| CT_max_ ~ svl + T_a_ |  |  |  | 90.81 | -41.40 |
|  | svl | 0.0451 | 0.6258 |  |  |
|  | T_a_ | 0.3721 | **<0.001** |  |  |
| CT_max_ ~ svl + T_e_ |  |  |  | 71.21 | -31.61 |
|  | svl | -0.0133 | 0.8073 |  |  |
|  | T_e_ | 0.4741 | **<0.001** |  |  |
| CT_max_ ~ mass + elev_midpoint |  |  |  | 92.18 | -42.09 |
|  | mass | -0.0923 | 0.8687 |  |  |
|  | elev_midpoint | -0.0022 | **0.0001** |  |  |
| CT_max_ ~ mass + T_a_ |  |  |  | 91.07 | -41.53 |
|  | mass | -0.0803 | 0.8824 |  |  |
|  | T_a_ | 0.3499 | **<0.001** |  |  |
| CT_max_ ~ mass + T_e_ |  |  |  | 70.8 | -31.4 |
|  | mass | -0.2099 | 0.5251 |  |  |
|  | T_e_ | 0.4682 | **<0.001** |  |  |

**Supplementary** **Table S6.** Linear regression models between CT_max_ and elevation estimated in species for which CT_max_ data were collected over a range >100 m in elevation. Elevational range refers to locations where experimental animals were captured in the field.

| **Species** | **df** | **Intercept** | **Slope** | **R^2^** | **P** | **Elevational range (m)** |
| --- | --- | --- | --- | --- | --- | --- |
| *Bryophryne cophites* | 46 | 25.57 | 0.0007 | 0.00 | 0.7437 | 379 |
| *Bryophryne hanssaueri* | 30 | 89.19 | -0.0190 | 0.25 | 0.0044 | 186 |
| *Bryophryne nubilosus* | 21 | 25.67 | 0.0006 | 0.00 | 0.7763 | 714 |
| *Noblella pygmaea* | 11 | 34.23 | -0.0025 | 0.07 | 0.4141 | 627 |
| *Noblella* sp. R | 8 | 36.60 | -0.0044 | 0.37 | 0.0816 | 375 |
| *Oreobates gemcare* | 31 | -24.81 | 0.0184 | 0.20 | 0.0108 | 155 |
| *Pristimantis carvalhoi* | 10 | 34.10 | -0.0047 | 0.69 | 0.0016 | 780 |
| *Pristimantis danae* | 82 | 34.90 | -0.0037 | 0.45 | 0.0000 | 855 |
| *Pristimantis ockendeni* | 13 | 30.15 | -0.0003 | 0.01 | 0.7053 | 865 |
| *Pristimantis pharangobates* | 77 | 26.97 | -0.0002 | 0.00 | 0.6704 | 1035 |
| *Pristimantis platydactylus* | 12 | 34.55 | -0.0032 | 0.61 | 0.0016 | 1270 |
| *Pristimantis reichlei* | 37 | 34.28 | -0.0061 | 0.57 | 0.0000 | 950 |
| *Pristimantis salaputium* | 10 | 36.26 | -0.0046 | 0.77 | 0.0004 | 1185 |
| *Pristimantis toftae* | 45 | 31.88 | -0.0015 | 0.16 | 0.0052 | 1492 |
| *Psychrophrynella* sp. P | 63 | 11.62 | 0.0063 | 0.16 | 0.0010 | 424 |
| *Psychrophrynella* sp. R | 9 | 29.23 | -0.0012 | 0.05 | 0.5471 | 371 |
| *Psychrophrynella usurpator* | 220 | 32.53 | -0.0014 | 0.03 | 0.0066 | 839 |
